# Outcomes of TB treatment in HIV co-infected TB patients in Ethiopia: a cross-sectional analytic study

**DOI:** 10.1186/s12879-016-1967-3

**Published:** 2016-11-04

**Authors:** Solomon Ahmed Ali, Thandisizwe R. Mavundla, Ribka Fantu, Tadesse Awoke

**Affiliations:** 1 Department of Health Studies, University of South Africa (UNISA), Pretoria, South Africa; 2 Addis Continental Institute of Public Health, Addis Ababa, Ethiopia; 3 University of Gondar, Gondar, Ethiopia; 4 Addis Ababa, Ethiopia

**Keywords:** TB treatment outcome, TB/HIV co-infection, Treatment success rate, Favourable outcomes, Unfavourable outcomes, Cure rate, Death rate

## Abstract

**Background:**

TB and HIV are the most prevalent communicable diseases of major public health importance in the populations of sub-Saharan African countries, and an estimated 30 % of HIV infected persons have dual infection with TB. TB is the leading cause of death in HIV infected individuals, and HIV co-infected TB patients have multiple individual, disease specific and treatment related factors that can adversely affect their treatment outcomes. There is lack of evidence on the individual patient outcomes of HIV co-infected TB patients who receive anti-TB treatment. It is relevant to understand the differential patient outcomes of HIV co-infected TB patients and identify the factors that are associated with these outcomes.

**Methods:**

A comparative analysis was done on the data from a random sample of 575 TB patients who were enrolled for TB treatment from January 2013 to December 2013 at eight health facilities in Ethiopia. A descriptive analysis was done on the data, and chi-square test and logistic regression analysis was conducted to compare TB treatment outcomes based on HIV status and to identify factors associated with these outcomes.

**Results:**

Out of a total of 575 TB patients enrolled into the study, 360 (62.6 %) were non-HIV infected, 169 (29.4 %) were HIV co-infected, and 46 (8 %) had no documented HIV status. The overall treatment success rate was 91.5 % for all the study participants. HIV co-infected TB patients have a treatment success rate of 88.2 % compared with 93.6 % for non-HIV infected study participants (*P* = 0.03). HIV co-infected TB patients had a significantly higher rate (11.8 % versus 6.4 %, *P* = 0.03) of unfavourable outcomes. The cure rate was significantly lower (10.1 % versus 24.2 %, *P* = 0.001) and the death rate higher in HIV co-infected TB patients (8.3 % versus 2.5 %, *P* = 0.014). Age and TB classification were significantly associated with treatment outcome. No association was found with starting ART, Cotrimoxazole prophylactic treatment or enrolment in HIV care.

**Conclusions:**

There is high TB treatment success rate among patients who have been treated for TB, but the treatment success rate and the cure rate in HIV co-infected TB patients is lower than that observed in non-HIV infected patients. Patients with advanced age and those with smear positive pulmonary TB have unfavourable treatment outcomes.

**Electronic supplementary material:**

The online version of this article (doi:10.1186/s12879-016-1967-3) contains supplementary material, which is available to authorized users.

## Background

Tuberculosis (TB) and Human Immunodeficiency Virus (HIV) are the most prevalent communicable diseases of major public health concern in the populations of sub-Saharan African (SSA) countries including Ethiopia. Co-infection with HIV and TB is very common with an estimated 30 % of HIV infected persons having dual infection with TB ([1]: [S202–203]). About 80 % of the total estimated disease burden of HIV associated TB is found in countries of SSA, and this part of the world has the “highest rates of cases and deaths per capita” attributable to TB disease [2]. TB is among the leading causes of morbidity and mortality in HIV infected individuals; and in patients with TB disease, co-infection with HIV significantly complicates both the diagnosis and management of TB disease ([1] [S202–203]).

Many of the countries most severely affected by the two epidemics are resource poor settings with weak health systems ([3]:S1–S3). Decreasing the burden of disease from both infections among the population and improving the health outcomes of the individual patients attending medical services is of paramount importance to mitigate the adverse impact from these conditions.

Early detection of TB disease and prompt initiation of effective treatment with potent anti-TB drug regimens is required to decrease morbidity and mortality from TB disease in HIV infected patients [4]. Furthermore, this approach is critically important for prevention of transmission of TB within the population.

Co-infection with HIV is associated with significantly increased likelihood of mortality from TB disease, and HIV co-infected TB patients have significantly lower cure rates and lower treatment success rates compared to non-HIV infected TB patients ([5]:222–226; [6]). HIV patients with active TB disease have a probability of dying of 15–20 % at 1 year while those without active TB disease have 7–8 % probability of dying at 1 year ([7]:1605–1612).

Ethiopia is one of the countries in SSA that is hardest hit by the TB and HIV epidemics. With an estimated national adult prevalence of 1.5 %, it is estimated that, in 2013, there were 734,048 adults and children infected with HIV in Ethiopia [8]. The incidence of TB was 258 cases per 100,000 populations in 2011 [2]. The prevalence of HIV among incident TB cases was found to be 17 % [2]. This indicates that there is a high burden from each disease and a considerable co-infection rate. However, there is lack of evidence on the individual patient outcomes of co-infected patients with active TB disease and receiving anti-TB treatment in Ethiopia. It is essential and timely to better understand how and why HIV co-infected TB patients have unfavourable outcomes, and comparison of the outcomes of co-infected TB patients with those without HIV infection is relevant to understand the magnitude of the problem.

TB/HIV co-infected patients have multiple individual, disease specific and treatment related factors that can adversely affect their treatment outcomes [9]. Identifying these factors would help to provide better medical care to these patients for improved health outcomes.

The objective of this study was to assess and explain the outcomes of TB treatment in TB patients with respect to their HIV status, and to identify the associated underlying factors that contribute to the occurrence of these outcomes.

## Methods

This study used cross-sectional study design.

The target population for this study was all patients diagnosed with active TB disease and put on anti-TB treatment regimens. The accessible population was all patients diagnosed with active TB disease and put on anti-TB treatment regimens in the selected health facilities for this study.

The data source for this study was the TB register at the TB clinics of the health facilities where all patients diagnosed with active TB disease are put on anti-TB treatment regimens and monitored throughout the course of their treatment. The TB registers in the TB clinics of these health facilities are used to record all relevant patient level and clinical information for the treatment and monitoring of TB patients, and also for reporting of patient level data. Therefore, the sampling frame for this study is the list of all TB patients enrolled (within the selected time frame for the study, which is January 2013–December 2013) in the selected health facilities at which the study is conducted.

### Operational definitions

([10]:52; [11]:43; [12]).

#### TB infection

Infection with mycobacterium tuberculosis bacilli.

#### Active TB disease

Presence of signs and symptoms of TB disease in an individual who is infected with mycobacterium tuberculosis bacilli.

#### Case of tuberculosis

A definite case of TB (a pulmonary TB case with one or more initial sputum smear examinations positive for acid-fast bacilli) or one in which a health worker (clinician or other medical practitioner) has diagnosed TB and has decided to treat the patient with a full course of TB treatment.

#### HIV infection

Infection with the Human Immune-deficiency Virus (HIV) that is confirmed by approved serologic tests.

#### TB/HIV co-infection

The presence of both TB and HIV infection in an individual patient.

#### TB treatment outcome

The final known status of a TB patient who was started on anti-TB treatment.

#### Cured

An initially smear-positive patient who is sputum smear-negative at, or 1 ‘month’ prior to, the completion of TB treatment and on at least one previous occasion (usually at the end of the 2^nd^ or 5^th^ month).

#### Treatment completed

A patient who completed anti-TB treatment without evidence of failure but for whom sputum smear or culture results are not available in the last month of treatment and on at least one previous occasion.

#### Treatment failure

A patient whose sputum smear or culture is positive at 5 months or later during treatment. Also included in this definition are patients found to harbour a multidrug-resistant (MDR) strain at any point of time during the treatment, whether they are smear-negative or -positive.

#### Defaulter

A patient who has been on treatment for at least 4 weeks and whose treatment was interrupted for 8 or more consecutive weeks.

#### Died

A patient who dies for any reason during the course of treatment.

#### Transfer out

A patient who started treatment and has been transferred to another reporting unit and for whom the treatment outcome is not known at the time of evaluation of treatment results.

#### Treatment success

The sum of patients who are declared ‘cured’ and those who have ‘completed’ treatment.

### Inclusion criteria

- Any individual who has been diagnosed with active TB disease based on the Ethiopian national TB guidelines recommendations
- He or she has been started on a course of anti-TB treatment regimen within the time frame of the study period

### Exclusion criteria

- Any individual who has taken less than 4 weeks of the course of anti-TB treatment regimen
- Individuals less than 15 years of age at initiation of anti-TB treatment

The study sites (health facilities) included in this study are selected based on convenience (geographic accessibility) and patient load among all health centres within the Addis Ababa city administration which is a major urban setting and the capital city of Ethiopia. Based on the current TB patient load at each site and the number of patients required to meet the sample size, eight health facilities are selected for conducting the study.

### Sampling

A simple random sampling method was used to enrol eligible study participants into the study. The number of study participants to be enrolled from each selected health facility was determined proportionally (based on patient load). A table of random numbers was used to select and enrol study participants into the study sample from the sampling frame at each health facility.

### Ethical issues related to sampling and data management

Prior to the commencement of the study, the final version of the study protocol had been submitted and ethical clearance obtained from the local IRB (Addis Ababa Regional Health Bureau Ethical Review Committee) and the UNISA College of Human Sciences (CHS) Health Studies Higher Degrees Committee (HSHDC).

### Sample size

The sample size calculation was done using the Epi Info 7 statistical software program. The prevalence of HIV in incident TB cases in Ethiopia was estimated to be 17 % [2]. The reported TB treatment success rate for year 2009 indicated in the 2011 WHO global TB report [6] shows a TB treatment success rate of 72 and 88 % for HIV co-infected and non-HIV infected TB patients respectively. Using these background data as inputs to the epi info 7 statistical software to calculate the sample size, 350 TB patients are needed (61 HIV co-infected, 289 non HIV infected) with a two sided confidence level of 95 % and a power of 80 %.

### Data management and analysis

A paper based data collection tool was used to abstract data from the TB registers. The collected data were transferred to an electronic format (Excel sheet) that was prepared to capture the study variables from the data collection tool. The data in the Excel electronic format were cleaned (See ‘Additional file 1’ for supporting data). Study participants without documented TB treatment outcome data (<1 % of the sample) were excluded from the study sample as their data cannot be part of the analysis. There was very limited information on patients who declined to be tested for HIV. These study participants with missing HIV status information were kept with the study sample to be part of the data analysis.

The investigator imported the cleaned data to SPSS statistical software (PASW Statistics 18) from the electronic Excel format. The continuous variable age was recoded into age groups for the purpose of the analysis. The treatment outcome was also recoded into two groups. The treatment outcomes ‘Cured’ and ‘Treatment completed’ were grouped together as favourable treatment outcomes or treatment success. The other treatment outcomes were put together as unfavourable treatment outcomes.

## Results

### Sample characteristics

The age of the study participants ranged from 15 to 90 years with 58.3 % in the 25–49 age range (Table 1), and male gender accounted for more than half of the study sample.

**Table 1:** Distribution of characteristics among study participants (*N* = 575)

	Number	Percent
Age group (years)		
15–24	151	26.3
25–34	184	32.0
35–49	151	26.3
50–64	60	10.4
64–90	29	5.0
Total	575	100.0
HIV status		
Non-reactive (HIV-negative)	360	62.6
Reactive (HIV-positive)	169	29.4
Missing (not done)	46	8.0
Total	575	100.0
Category of TB treatment		
Default	2	0.3
Failure	1	0.2
New	488	84.9
Other	51	8.9
Relapse	17	3.0
Transfer-in	16	2.8
Total	575	100.0

Among the 575 study participants enrolled in the study, 529 (92 %) of them had a documented HIV test offered and performed at or prior to the beginning of their course of anti-TB treatment, and almost one-third of the study participants have documented HIV co-infection (i.e. HIV positive) as shown in Table 1.

Most of the study participants were diagnosed with pulmonary tuberculosis (55.8 %) and a quarter of all cases of TB were found to have smear-positive pulmonary tuberculosis. The majority of the study participants were new TB patients who started their TB treatment at the health facility where they have completed their course of TB treatment (Table 1).

**Fig. 1:**
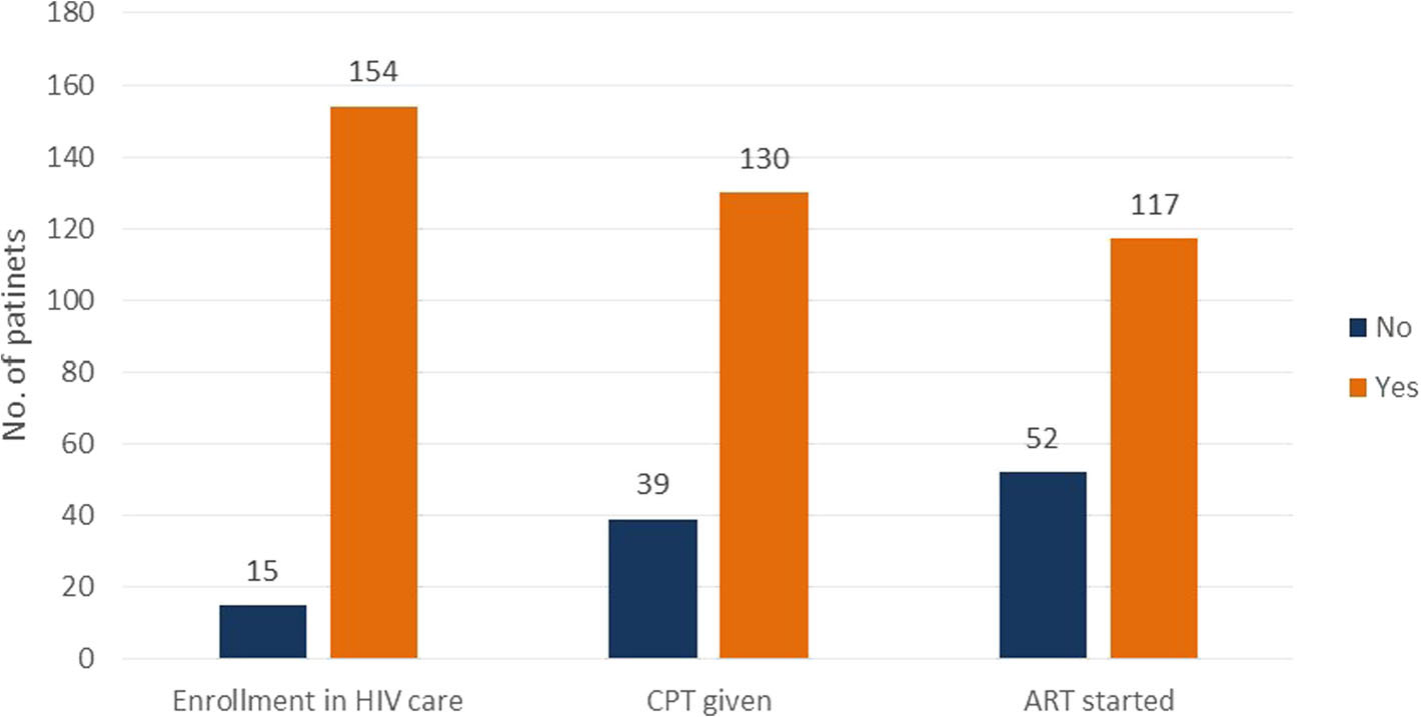
Status of HIV co-infected study participants by medical care services received

More than 90 % of the study participants with TB/HIV co-infection were enrolled in HIV care, and most of them provided with Anti-Retroviral Treatment (ART) and/or co-trimoxazole prophylactic treatment (Fig. 1, *N* = 169).

### Treatment outcomes of the study participants

**Fig. 2:**
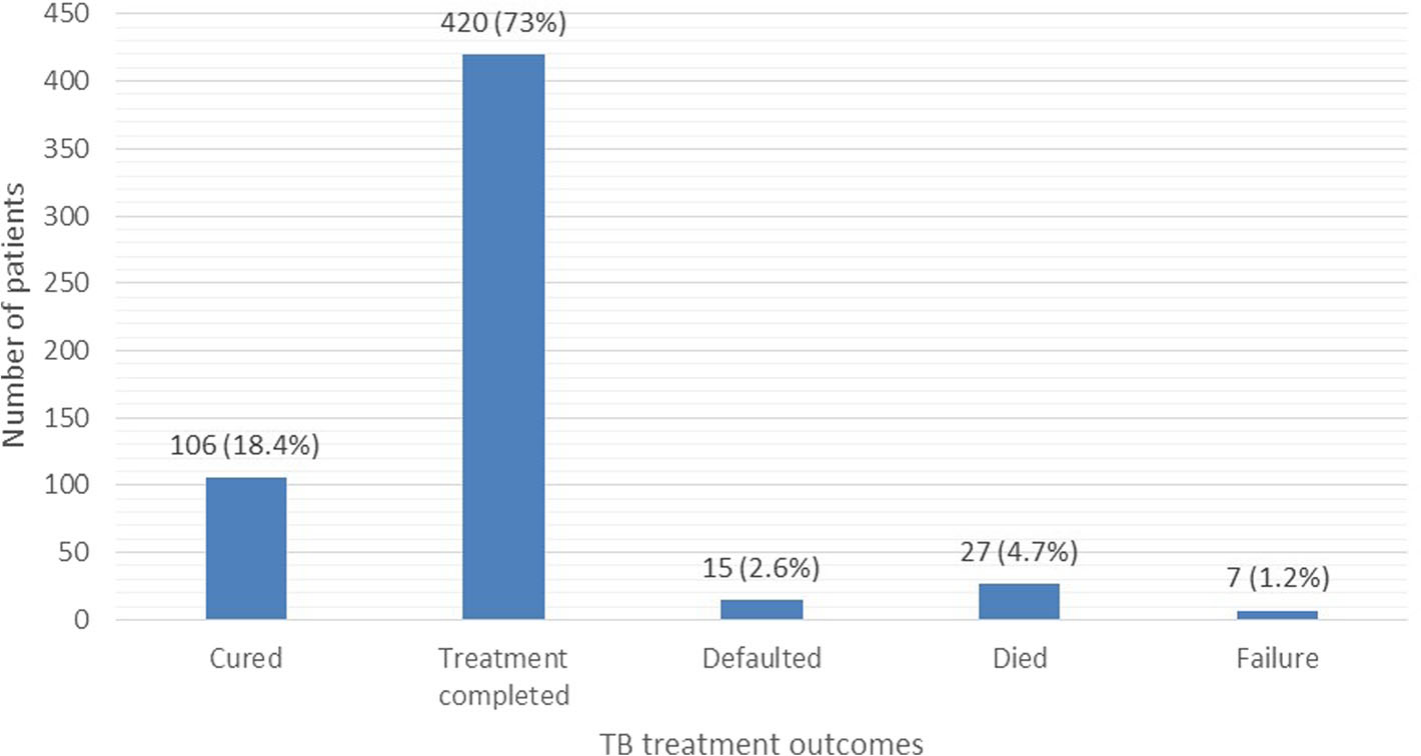
Outcomes of TB treatment among the study participants

Analysis of the study data demonstrates that the outcome of TB treatment for the study participants shows a very high overall treatment success rate (91.5 %) defined as either ‘Cured’ or ‘Treatment completed’ after a course of anti-TB treatment (Fig. 2, *N* = 575). The treatment success rate was 93.6 % among non-HIV infected TB patients in contrast to 88.2 % among HIV co-infected TB patients (Table 2) which was statistically significant (*P* = 0.03, Chi-Square test).

**Table 2:** Comparison of TB treatment success based on HIV status (*N* = 529)

TB treatment outcome	HIV status		Total
	HIV negative	HIV positive	
Unfavourable outcome	23 (6.4 %)	20 (11.8 %)	43 (8.1 %)
Favourable outcome (treatment success)	337 (93.6 %)	149 (88.2 %)	486 (91.9 %)
Total	360 (100.0 %)	169 (100.0 %)	529 (100.0 %)

### TB treatment outcomes in relation to HIV status

**Fig. 3:**
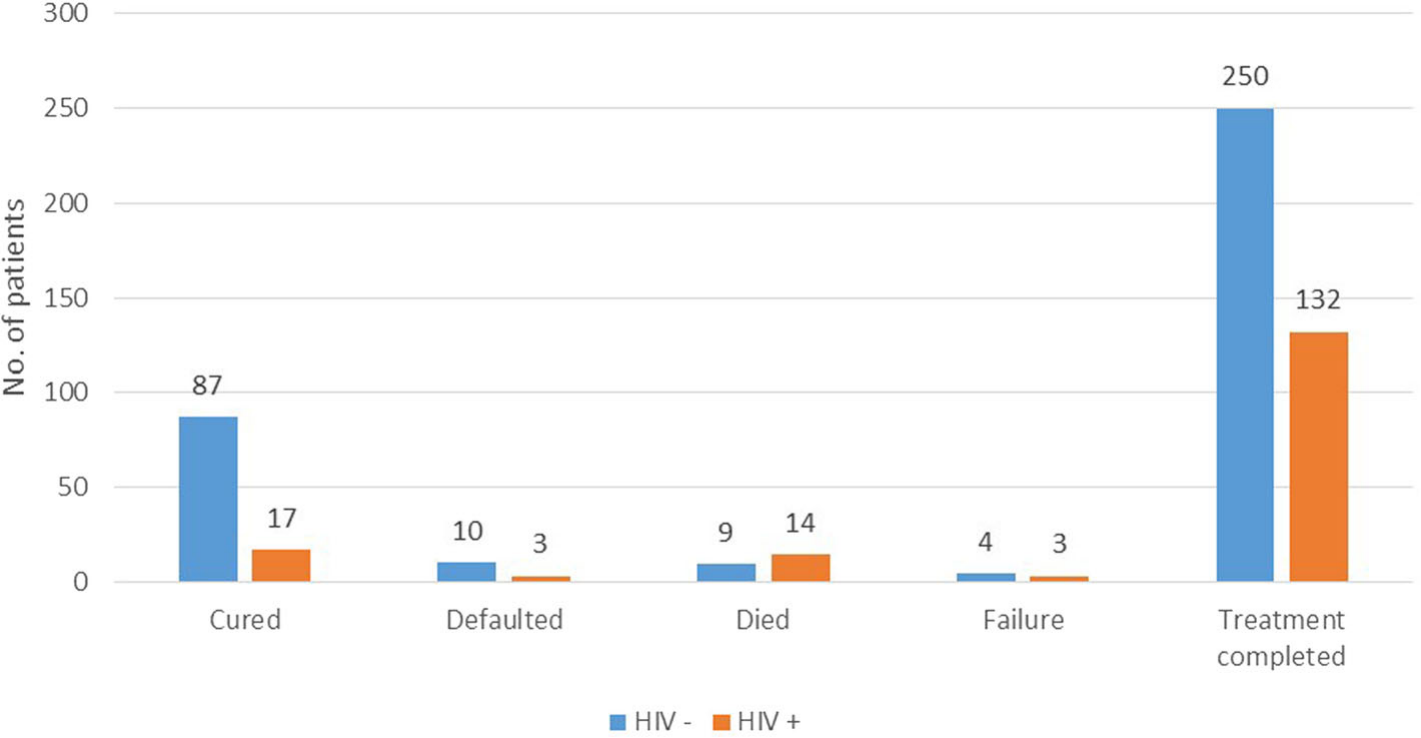
Comparison of TB treatment outcomes and HIV status

In this study, HIV co-infected TB patients have a treatment success rate of 88.2 %, and a higher rate (11.8 %) of unfavourable treatment outcomes (defined as death, default or failure) compared to those without underlying HIV infection (6.4 %) as shown in Table 2. These differences in treatment outcomes were statistically significant (*P* = 0.03, Chi-Square test). Figure 3 shows the comparison of all the TB treatment outcomes in relation to HIV status.

The cure rate among HIV co-infected TB patients was significantly lower and the death rate significantly higher (Table 3) than that among those without underlying HIV infection (10.1 % versus 24.2 % and 8.3 % vs 2.5 %, respectively).

**Table 3:** Comparison of TB treatment outcomes based on HIV status (*N* = 529)

Treatment outcome	HIV −	HIV +	OR	95 % CI	*P* value
Cured	87 (24.2 %)	17 (10.1 %)	2.702	1.542–4.735	0.001
Defaulted	10 (2.8 %)	3 (1.8 %)	1.76	0.476–6.505	0.397
Died	9 (2.5 %)	14 (8.3 %)	0.339	0.143–0.805	0.014
Failure	4 (1.1 %)	3 (1.8 %)	0.704	0.155–3.192	0.649
Treatment completed	250 (69.4 %)	132 (78.1 %)			
Total	360 (100 %)	169 (100 %)			

### Other characteristics associated with treatment outcome

Further analysis of the study data was performed to identify other factors (variables) that potentially affect the outcome of TB treatment in the study population. This analysis showed that age and TB classification are significantly associated with TB treatment outcome. Table 4 provides a detailed account of the results of the analysis. No association was observed between TB treatment outcome and receiving other medical care services like starting ART, co-trimoxazole prophylactic treatment or enrolment in HIV care.

**Table 4:** Binary regression analysis of the association of patients’ characteristics with TB treatment outcome

Variable	Value	TB treatment outcome		Total	OR	95 % CI	*P* value
		No success	Success				
Age	15–24	9 (6.0 %)	142 (94.0 %)	151	0.243	0.079–0.747	0.014
	25–34	15 (8.2 %)	169 (91.8 %)	184	0.34	0.120–0.965	0.043
	35–49	10 (6.6 %)	141 (93.4 %)	151	0.272	0.090–0.820	0.021
	50–64	9 (15 %)	51 (85 %)	60	0.676	0.215–2.124	0.503
	65–90	6 (20.7 %)	23 (79.3 %)	29			
Gender	F	22 (8.3 %)	242 (91.7 %)	264	0.956	0.531–1.722	0.881
	M	27 (8.7 %)	284 (91.3 %)	311			
TB classification	EP	17 (6.7 %)	236 (93.3 %)	253	0.436	0.22–0.863	0.017
	P/neg	12 (6.7 %)	168 (93.3 %)	180	0.432	0.204–0.917	0.029
	P/pos	20 (14.2 %)	121 (85.8 %)	141			
Enrollment in HIV care	No	3 (20.0 %)	12 (80.0 %)	15	2.015	0.516–7.864	0.313
	Yes	17 (11.0 %)	137 (89 %)	154			
Co-trimoxazole given	No	7 (17.9 %)	32 (82.1 %)	39	1.969	0.725–5.344	0.184
	Yes	13 (10.0 %)	117 (90.0 %)	130			
ART started	No	8 (15.4 %)	44 (84.6 %)	52	1.591	0.608–4.161	0.344
	Yes	12 (10.3 %)	105 (89.7 %)	117			
HIV status	Negative	23 (6.4 %)	337 (93.6 %)	360	0.508	0.271–0.954	0.035
	Positive	20 (11.8 %)	149 (88.2 %)	169			

Looking at HIV co-infected patients separately, the analysis shows that there were 37 patients (22 %) who had been on ART for more than 6 months when they developed active TB disease. The occurrence of active TB disease in those HIV patients who have been on ART for more than 6 months makes these patients suspected for the possibility of the emergence of treatment failure on the ART regimen that these patients were receiving. This might have dampened the positive effect of ART on TB treatment outcomes. However, a separate analysis comparing the TB treatment outcomes among those who developed active TB disease while taking ART for more than 6 months with the other study participants (who started ART after TB diagnosis or were taking ART less than 6 months when developing active TB disease) failed to show a significant difference in TB treatment outcome between them (OR = 0.613 [0.218–1.726]; *P* = 0.354).

For those factors that showed significant association on initial analysis, multiple regression analysis was done. The results show that these factors are still significantly associated with outcomes of TB treatment (Table 5).

**Table 5:** Multiple regression analysis of factors associated with TB treatment outcome

Variable	Value	TB treatment outcome		Total	OR	AOR	95 % CI	*P* value
		No success	Success					
Age	15–24	9 (6.0 %)	142 (94.0 %)	151	0.243	0.150	0.041–0.546	0.004
	25–34	15 (8.2 %)	169 (91.8 %)	184	0.34	0.185	0.055–0.619	0.006
	35–49	10 (6.6 %)	141 (93.4 %)	151	0.272	0.126	0.034–0.468	0.002
	50–64	9 (15 %)	51 (85 %)	60	0.676	0.389	0.104–1.457	0.161
	65–90	6 (20.7 %)	23 (79.3 %)	29				
TB classification	EP	17 (6.7 %)	236 (93.3 %)	253	0.436	0.322	0.149–0.697	0.004
	P/neg	12 (6.7 %)	168 (93.3 %)	180	0.432	0.329	0.146–0.744	0.008
	P/pos	20 (14.2 %)	121 (85.8 %)	141				
HIV status	Negative	23 (6.4 %)	337 (93.6 %)	360	0.508	0.367	0.178–0.757	0.007
	Positive	20 (11.8 %)	149 (88.2 %)	169				

## Discussion

In this study, more than half (58.3 %) of the study participants were in the age groups between 25 and 49 years. Among the 575 study participants who were enrolled into the study sample, 529 (92 %) have a documented HIV test result, among whom about one-third (1/3) were HIV-positive. These findings are much higher than that reported for Ethiopia for 2013 with just 71 % of TB patients having a documented HIV status and only 11 % of those with documented status being HIV-positive [13].

Pulmonary tuberculosis was the most common presentation of TB disease among the study participants (55.8 %), and a quarter of all cases of TB were found to have smear-positive pulmonary tuberculosis (P/pos). Extra-Pulmonary (EP) cases accounted for 44 % of the total.

Among those study participants who had co-infection with HIV (*N* = 169), a high proportion of them were receiving care and treatment services for HIV with 91.1 % enrolled in HIV care, 76.9 % received co-trimoxazole prophylactic treatment and 69.2 % regularly receiving ART.

There was a very high rate of treatment success observed in this study. Overall, treatment success rate was 91.5 % for all the study participants. The success rate was 88.2 % among HIV co-infected TB patients in contrast to 93.6 % for all non-HIV infected TB patients. HIV co-infected TB patients have a higher rate (11.8 %) of unfavourable treatment outcomes (defined as death, default or failure) compared to those without underlying HIV infection (6.4 %). Other similar studies conducted in high prevalence settings showed that the highest treatment success rate (85 %) is observed in HIV non-infected TB patients and overall success rate (for both groups combined) is 77 % ([14]:521–528; [15]:157). From global TB reports by the WHO, the best treatment success rates (95 %) were reported from China among all new TB patients [13]. The reported treatment success rate for Ethiopia among all new TB cases in 2012 was 91 %, which is the highest reported yet for this country and showing a progressively improving trend over the years [13]. The same report indicates that the treatment success rate for Ethiopia was less than 80 % in the years prior to the year 2011.

The cure rate among HIV co-infected TB patients was significantly lower than among those without underlying HIV infection (10.1 % versus 24.2 %); whereas, the death rate was higher in HIV co-infected TB patients (8.3 % versus 2.5 %). These findings are consistent with the expected outcomes among HIV co-infected patients including observations from studies and programme reports. According to the global TB report [13], globally in 2012, HIV co-infected TB patients had 74 % favourable treatment outcomes compared with 88 % for HIV-negative TB patients, though the difference was smaller in the African region (75 % in co-infected versus 83 % in non-HIV infected). In this same report, there were very similar findings regarding TB patient mortality which showed the proportion of TB patients that died during treatment was more than three times higher among HIV-co-infected TB patients (11 % versus 3.4 %).

In addition to HIV status, age and TB classification were significantly associated with TB treatment outcome in this study. TB patients in the age group above 65 years of age had a higher likelihood of “unfavourable outcome” than those in the other age groups. Other studies have also demonstrated the association of adverse TB treatment outcomes and advanced age. A study conducted in South India ([16]:e67288) showed that TB treatment outcomes were poor among older TB patients and there was an increased risk of unfavourable outcomes in those above the age of 60 years. Another study in Delhi, India also showed a significantly higher rate of death and TB treatment failure among TB patients older than 65 years of age ([17]:83).

Lower TB treatment success was observed among those patients with smear positive pulmonary TB. This is consistent with the results of other studies in similar settings. A study in India demonstrated that TB disease classification is significantly associated with TB treatment outcome and patients under ‘Pulmonary’ TB classification had “unfavourable outcome” ([18]:e21008). Another Nigerian study showed that those TB patients with smear positive status at diagnosis (i.e. pulmonary positive TB classification) have more adverse TB treatment outcomes ([19]:210).

The unexpected finding in this study was the absence of association between TB treatment outcome and the other medical care services received by HIV co-infected TB patients, namely starting ART, Cotrimoxazole prophylactic treatment and enrolment in HIV care. These findings are in contrast to results of other studies from other settings which showed that TB patients who were put on ART had more successful treatment outcomes than those not taking ART ([20]:e56248; [18]:e21008). A similar study in HIV co-infected TB patients in Italy showed that there is a marked reduction in death rate in patients who were taking ART during tuberculosis treatment; but patients who were already on ART when they were diagnosed to have tuberculosis had higher risk of death ([21]:1).

Additional analysis of the data from our study showed that 22 % of HIV co-infected TB patients were taking ART for more than 6 months when they were diagnosed to have active TB disease and started on TB treatment. This finding could be indicative of the emergence of treatment failure on the ART regimen that these patients were taking. This could have contributed to dampen the positive effect of ART on TB treatment outcomes. However, no difference in treatment outcome was observed between those who developed active TB disease while taking ART for more than 6 months and the other study participants. Another point to consider would be that many HIV co-infected TB patients might have been diagnosed too late in the course of their illnesses (advanced stages of HIV) to have any significant benefit from HIV care and treatment services.

### Limitations

A limitation of this study could be that all the sites are within Addis Ababa which is the capital and the largest town in the country. Patients from these facilities might have a different profile from patients residing in other parts of the country.

Another limitation is that the data collected is retrospective secondary data. For some study participants, there were issues with missing and/or inaccurate data. In addition, the data source (which was the standard TB registers) did not capture detailed information on HIV co-infected TB patients’ clinical and immunological status (ex. CD4 count, viral load), and other HIV related services (ex. ARV regimens, adherence).

Prospective quantitative studies and qualitative studies are required to further explore additional patient level and service delivery factors that may affect the outcome of HIV co-infected TB patients.

## Conclusions

In this study, we observed that there was a very high rate of TB treatment success, even among HIV co-infected TB patients. Nevertheless, there were more unfavourable outcomes among the HIV co-infected TB patients, and the provision of HIV treatment and care services (ART, co-trimoxazole etc.) was not found to have significant effect on TB treatment outcome. Further prospective and qualitative studies will be important to assess the reason why receiving HIV care and treatment services had been seen to have no effect on TB treatment outcomes of HIV co-infected TB patients. Delayed identification of HIV and/or TB might have negatively affected the outcomes of these patients. In addition, it is worthwhile to investigate individual patient level factors and/or treatment regimen related factors for TB patients with advanced age and those with smear positive pulmonary TB, as more unfavourable outcomes were observed among these patients.

## Additional file

Additional file 1: Cleaned data on Outcomes of TB treatment Ethiopia. This is the cleaned set of data of the 575 individual study participants that were included in the analysis. It shows the age and gender of the participants, their clinical characteristics and medical care received, and their individual health outcomes. (XLSX 55 kb)

## Abbreviations

ART: Anti-Retroviral Treatment
EP: Extra pulmonary tuberculosis
HIV: Human Immunodeficiency Virus
P/pos: Smear Positive Pulmonary Tuberculosis
SSA: sub-Saharan Africa
TB: Tuberculosis
UCSF: University of California San Francisco
UNISA: University of South Africa
WHO: World Health Organization
